# *In vitro* models for head and neck cancer: Current status and future perspective

**DOI:** 10.3389/fonc.2022.960340

**Published:** 2022-08-03

**Authors:** Christian R. Moya-Garcia, Hideaki Okuyama, Nader Sadeghi, Jianyu Li, Maryam Tabrizian, Nicole Y. K. Li-Jessen

**Affiliations:** ^1^ Department of Biomedical Engineering, McGill University, Montreal, QC, Canada; ^2^ School of Communication Sciences and Disorders, McGill University, Montreal, QC, Canada; ^3^ Department of Otolaryngology – Head & Neck Surgery, Kyoto University, Kyoto, Japan; ^4^ Department of Otolaryngology – Head and Neck Surgery, McGill University, Montreal, QC, Canada; ^5^ Research Institute of McGill University Health Center, McGill University, Montreal, QC, Canada; ^6^ Department of Mechanical Engineering, McGill University, Montreal, QC, Canada; ^7^ Faculty of Dental Medicine and Oral Health Sciences, McGill University, Montreal, QC, Canada

**Keywords:** head and neck cancer, tumor micoenvironment, 3D cancer models, spheroids, organotypic models, microfluidic devices, drug screening

## Abstract

The 5-year overall survival rate remains approximately 50% for head and neck (H&N) cancer patients, even though new cancer drugs have been approved for clinical use since 2016. Cancer drug studies are now moving toward the use of three-dimensional culture models for better emulating the unique tumor microenvironment (TME) and better predicting *in vivo* response to cancer treatments. Distinctive TME features, such as tumor geometry, heterogenous cellularity, and hypoxic cues, notably affect tissue aggressiveness and drug resistance. However, these features have not been fully incorporated into *in vitro* H&N cancer models. This review paper aims to provide a scholarly assessment of the designs, contributions, and limitations of *in vitro* models in H&N cancer drug research. We first review the TME features of H&N cancer that are most relevant to *in vitro* drug evaluation. We then evaluate a selection of advanced culture models, namely, spheroids, organotypic models, and microfluidic chips, in their applications for H&N cancer drug research. Lastly, we propose future opportunities of *in vitro* H&N cancer research in the prospects of high-throughput drug screening and patient-specific drug evaluation.

## Introduction

Cancer drug research and development (R&D) are considered as one of the most expensive expenditures among drug development as compared to that of all other diseases (1). The global spending on oncology drugs reached $164 billion in 2020 and an estimated $269 billion by 2025 even as annual growth rates ease to approximately 10% (2). Mailankody and Prasad from National Cancer Institutes in the United States critically pointed out that new cancer drugs may not necessarily help to increase the survival rate in cancer patients despite the expensive investments in cancer drug R&D (3). In 2016, the Food and Drug Administration (FDA) approved the chemotherapy drug hydroxyurea for the treatment of locally advanced head and neck (H&N) cancer as well as the immunotherapy drugs pembrolizumab and nivolumab for recurrent/metastatic H&N cancer (4). Since, the role of these three drugs in the H&N cancer primary treatment has not been properly elucidated, the 5-year overall survival of H&N cancer patients remains less than 50% (5) with 30% of them experiencing cancer relapse and resistance to treatment (6).

The R&D pipeline for new drug discoveries starts with *in vitro* models, followed by preclinical/animal testing and clinical trials. *In vitro* platforms often represent a first milestone to reach the evaluation of drug cytotoxicity, dose, resistance, and sensitivity as well as the identification of the target molecular mechanisms of prognostic markers (7). Specific to cancer drug screening and discovery, *in vitro* models are often designed to mimic the tumor microenvironment (TME) of interest (8, 9). For instance, an overexpression of epithelial growth factor receptors (EGFRs) were noted in almost 90% of patients with H&N tumors (10, 11). To reflect this environment, in one of the very early *in vitro* studies with H&N squamous cell carcinoma cultures collected from larynx, retromolar trigone, cervical lymph node, and the floor of mouth, the inhibition of the EGFR was assessed by incorporating two anti-EGFR monoclonal antibodies (MAbs 425 and 528) based on *in vitro* models (12). Cell viability results showed that the two anti-EGFR antibodies reduced cancer cell growth by up to 97% compared to healthy mucosal epithelial cells after a 5-day exposure. Further, *in vitro* and *in vivo* studies on monoclonal antibodies against EGFR led to the discovery of cetuximab, which was approved by the FDA for colon cancer treatment in 2004 and in 2011 for the treatment of recurrent/metastatic H&N cancer (13).

The recent evolution of *in vitro* cancer models has been focused on emulating the tissue-specific TME as much as possible to recapitulate drug resistance and uptake in specific tumor tissues. Advances in spheroid/organoid bioengineering and their culturing methods, as well as microfluidic technologies, are harnessed to enable physiologically and clinically relevant *in vitro* cancer models. Distinctive TME features, namely, three-dimensional (3D) tumor geometry, heterogeneous cell populations, and fenestrated tumor vasculature, have been incorporated into *in vitro* models, such as breast (14), lung (15), and liver (16) cancers. However, tissue-specific TME features have not been fully applied to *in vitro* H&N cancer model designs, which might explain the slow advancement of effective drug discovery and longitudinal drug evaluation for H&N cancers.

**Figure 1:**
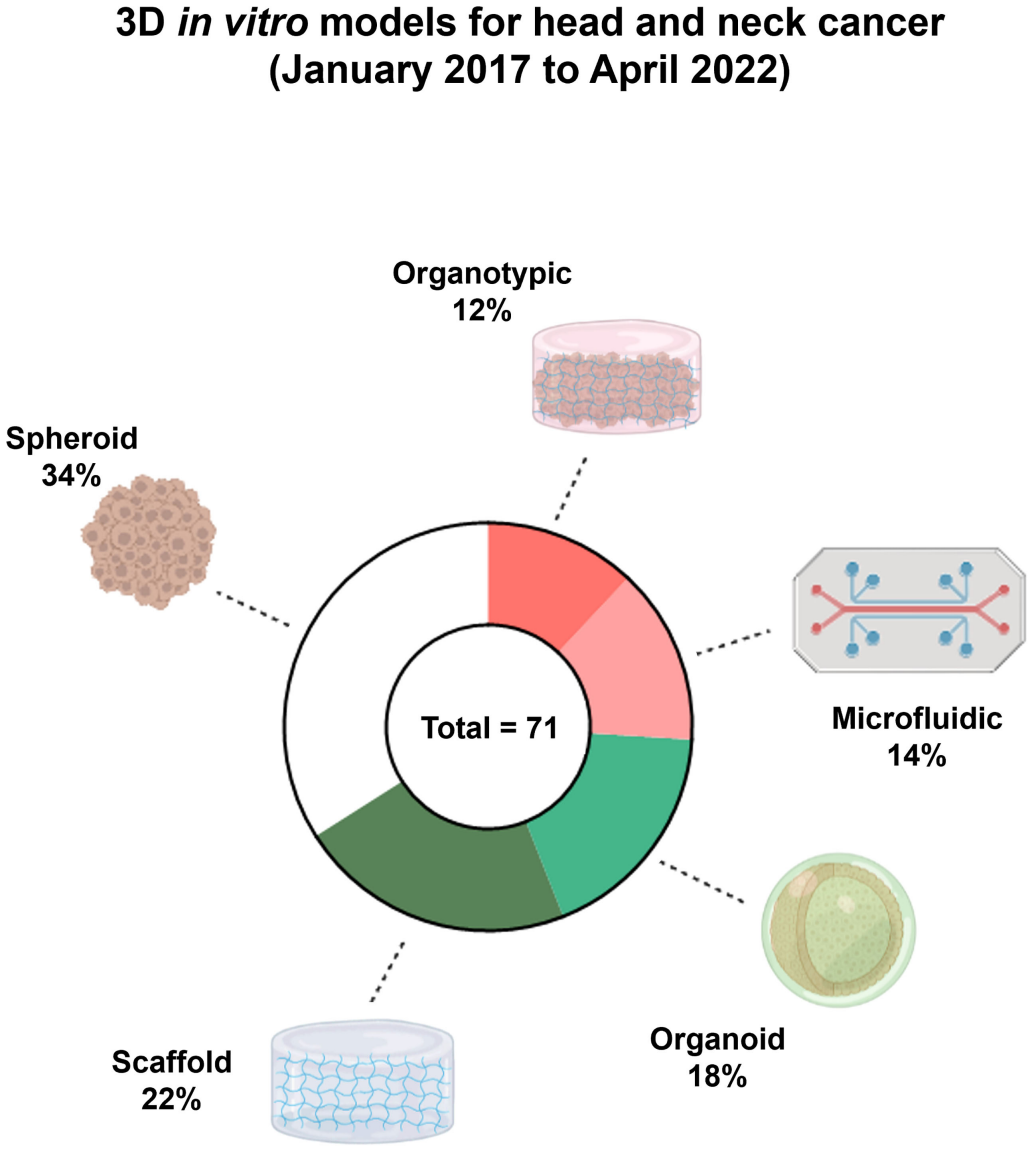
Culturing models in head and neck (H&N) cancers. Pie graph of published articles between 2017 and 2022 using the NCBI PubMed^®^. Related publications of three-dimensional (3D) *in vitro* models in H&N cancer with spheroids being the most abundant type of culture model. Figure created with BioRender.com and GraphPad Prism 9.3.1.

To survey the current implementation of 3D *in vitro* models for H&N cancer, we performed a search for original research papers published on The National Center for Biotechnology Information (NCBI) PubMed^®^ between January 2017 and April 2022 using the following combined terms, namely, “*head and neck cancer*,” “*spheroid*,” “*organoid*,” “*microfluidic*,” and “*organotypic*” (**Figure 1**). The search generated 71 research studies. Spheroid cultures (34%; N = 24) and scaffold models (22%; N = 16) were the two most common 3D culture models in H&N cancer research.

To understand the uptake of 3D *in vitro* models for H&N cancer drug discovery, a search was performed on the original studies of 12 common cancers including H&N (17) published on NCBI PubMed^®^ between January 2017 and April 2022 using the following combined terms: “*in vitro*”, “*drug discovery*”, “*breast*”, ‘‘*lung*’’, ‘‘*colorectal*’’, ‘‘*glioblastoma*’’, ‘‘*prostate*’’, ‘‘*melanoma*’’, ‘‘*lymphoma*’’, ‘‘*pancreatic*’’, ‘‘*cervical*’’, ‘‘*head and neck’’*, “*thyroid*”, “*oral*”, “*laryngeal*”, ‘‘*bladder*’’, ‘‘*renal*’’, and “*cancer*”. The search generated 489 results. Among the 12 organs searched, approximately 27.6% (N = 135) were related to breast cancer while only 2.2% (N = 11) were associated with H&N cancer. Further search on drug discovery–related publication for H&N cancer showed that only 3 out of the 11 results used 3D *in vitro* models. In other words, approximately 4.2% [(3 out of 11)/71] of 3D *in vitro* models were applied in the study of cancer drug discovery. The aforesaid statement described the need for more H&N cancer research using advanced 3D *in vitro* models instead of conventional 2D cultures for developing new anticancer drugs.

In this paper, we review the unique TME characteristics in H&N cancers and their relevance to the tumor tissue aggressiveness and drug resistance. We present the design principles of *in vitro* models to mimic key TME features relevant to H&N cancer. We then report on several state-of-the-art culturing models, namely, spheroids, 3D scaffolds, organotypic models, and microfluidic devices that have contributed to the H&N cancer therapeutic R&D. Finally, we provide a perspective on more reproducible and robust *in vitro* H&N cancer models for high-throughput drug screening and patient-specific drug development.

## Tumor microenvironment in head and neck cancers

**Figure 2:**
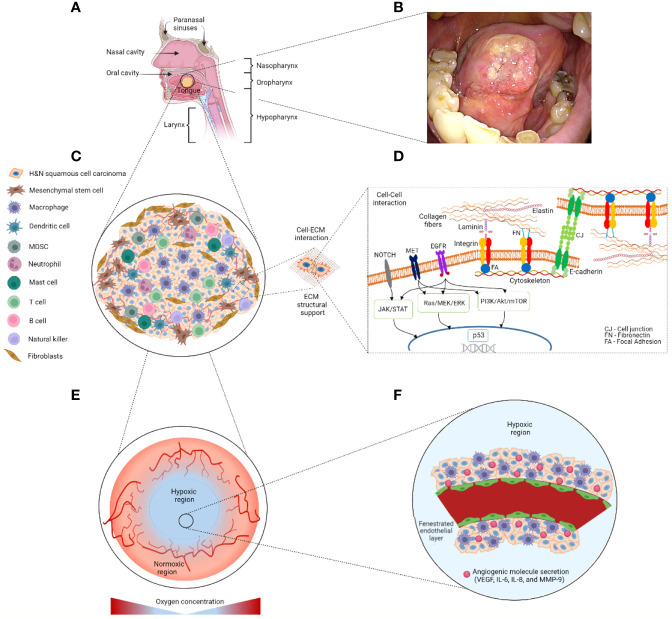
Schematic representation of the potential tumor location and tumor microenvironment (TME) in H&N cancer. **(A)** H&N cancer may be found at oral, nasal, and paranasal cavities, larynx, and pharynx anatomical sites. **(B)** Clinical image of stage 2 tongue cancer (<4 cm) provided by Drs. Yo Kishimoto and Hideaki Okuyama’s research team at the Kyoto University Hospital with patient’s consent. **(C)** Heterogeneous cell populations are resided within an H&N squamous cell carcinoma. Stromal cells including mesenchymal stem cells and fibroblasts are commonly found in the outer layer of the tumorous body. Tumor-infiltrating immune cells including macrophages and T cells among others are found within the tumor. **(D)** The extracellular matrix provides structural support and biochemical cues to the TME *via* cell–cell/–ECM interactions. Mutation of pathways PI3K/Akt/mTOR, TP53, NOTCH, EGFR, JAK/STAT, Ras/MEK/ERK, and MET relate to H&N cancer development. **(E)** The hypoxic region is located at the center of the tumor, which is characterized by aberrant vasculature. **(F)** This fenestrated vasculature hampers the proper supply of nutrients, oxygen, and therapeutics. ECM, extracellular matrix; IL, interleukin; MDSC, myeloid-derived suppressor cell; MMP, metalloproteinase; VEGF, vascular epithelial growth factor. Figure created with BioRender.com.

A typical TME in H&N cancer is heterogeneously composed of neoplastic cells, endothelial cells, and fibroblasts, as well as tumor-infiltrating immune cells from the mucosae of the oral, nasal and paranasal cavities, larynx, and pharynx (6, 17) (**Figure 2**). Approximately 90% of H&N cancer cells are considered as squamous cell carcinomas (6, 18). The H&N carcinomas present an air–liquid interface conformation since the apical TME is in contact with the air from the cavity lumen whereas the basal TME interacts with blood (6, 18, 19). In particular, these fish scale–like/squamous epithelial neoplastic cells exhibit an aggressive abnormal cell proliferation crossing the boundaries of surrounding cells in concert with endothelial cells and fibroblasts (18). Extracellular matrix (ECM) proteins as collagen, elastin, fibronectin, and laminin provide a structural support that plays a part in cell adhesion and migration in the TME of H&N (19).

H&N squamous cell carcinomas may present oncogenes associated with human papillomavirus (HPV) infection (18, 20), largely p16 followed by p18 genes (20). A classification of H&N squamous cell carcinoma relies on the presence of HPV-associated oncogenes that are normally referred to as HPV^+^ or HPV^-^ H&N cancer (20). In particular, the mutation and down- or upregulation of molecular mechanisms such as PI3K/Akt/mTOR (mammalian target of rapamycin), TP53, NOTCH, EGFR, JAK/STAT, Ras/MEK/ERK, and MET pathways are found to be associated with the progression of H&N squamous cell carcinoma (20) (**Figure 2D**). For example, the PI3K/Akt/mTOR pathway is upregulated in more than 90% of H&N squamous cell carcinomas, resulting in an increased resistance to chemotherapy and radiotherapy and cancer progression (21).

Similar to other cancer progressions, in H&N cancer, epithelial, mesothelial, and endothelial cells shift from a basal to mesenchymal phenotype that allows these cells to acquire mobility and protect tumor cells from anoikis, a programmed cell death (19). These phenomena are commonly known as epithelial, mesothelial, and endothelial mesenchymal transitions, respectively. Cancer-associated fibroblasts may differentiate from resident fibroblasts and from epithelial, mesothelial, and endothelial cells during respective mesenchymal transitions. Cancer-associated fibroblasts play important roles in tumor growth and maintenance through secreting autocrine and paracrine signaling molecules such as IL-1α, IL-1β, IL-6, IL-33, HGF, VEGF, TNF-α, TGF-β, CCL-2, CXCL-12, CXCR-4, MMP-2, and Snail (17, 19, 22). Cancer-associated fibroblasts in concert with endothelial cells secrete EGF that enhances tumorous motility and stemness (23, 24). In addition, stromal cells such as fibroblasts produce ECM proteins (e.g., collagen, elastin, and fibronectin) that create the fibrous architectural conformation of the tumorous body (19, 25). This structural fibrous network contributes to cell adhesion, cell proliferation, and cell migration, which, in turn, leads to tumor progression and reduced response to treatment (18, 19, 25).

Specific to the H&N cancer, the TME aggressiveness and resistance to treatment are linked to two primary mechanisms, namely, the dysregulation of the immune system and tumor hypoxia (20). With respect to the dysregulated immune system, a plethora of immune cells including T cells (cytotoxic and regulatory phenotypes), B cells, natural killers, tumor-associated macrophages (anti- and pro-tumor phenotypes), tumor-associated neutrophils, myeloid-derived suppressor cells, and mast cells are found within the TME of H&N tumors (6, 26). Checkpoint markers, including programmed cell death 1 (PD-1) and its ligand PD-L1, were found upregulated on exhausted T cells and myeloid-derived suppressor cells in the H&N TME (6). As a result, two PD-1 inhibitor drugs, pembrolizumab and nivolumab, were developed and approved for H&N cancer treatment in 2016, for unresectable and cisplatin-resistant recurrent/metastatic H&N cancer (4, 27, 28).

Tumor hypoxia is another well-recognized factor contributing to the aggressive tumor behavior and drug resistance in H&N cancer (19, 20, 29). The fenestrated tumor vessels result in aberrant tumor blood flow to the underperfused areas of the solid tumor (**Figures 2E**, **F**). In particular, oxygen, nutrients, and drugs are restricted to reach the cells in certain tumor areas, leading to some high-level hypoxic regions within the TME (18, 20). Pro-tumor/anti-inflammatory macrophages are reported to secrete excessive angiogenic cytokines such as VEGF, IL-6, IL-8, CCL-2, and MMP-9, which results in aberrant angiogenesis and the hypoxic H&N-specific TME *in vitro* and *in vivo* (6, 17, 18, 30, 31).

## Design principles of *in vitro* head and neck cancer models

Like many other *in vitro* models mimicking the TME, a representative *in vitro* H&N tumor model is expected to sufficiently recapitulate: (I) a 3D tumor-like geometry for cell–cell and cell–ECM interactions; (II) the heterogeneous cell types such as squamous cell carcinomas, stromal, and immune cells in the TME; and (III) the aberrant and fenestrated vasculature for the high-level hypoxic TME (**Figure 2**). These principles are further elaborated in the following paragraphs.

## Three-dimensional tumor geometry

Tumors are 3D sphere-like solid structures with unique physical and biochemical boundaries, in which they need to be considered for cancer drug screening and evaluation. First, the physical geometry of the tumor affects drug disposition, diffusion, and absorption (32–34). For instance, the flat two-dimensional (2D) monolayer geometry exposes the drug application to the entire cell monolayer, making the cells more susceptible to the applied drug compared to that of 3D geometry (35, 36). Advanced *in vitro* cancer models have incorporated 3D spherical geometries to make the drug diffusion and uptake by cellular targets more similar to the *in vivo* settings of solid H&N tumors. Second, the 3D tumor geometry is a key parameter in the organization of cell membrane receptors and the remodeling of ECM constituents, which, in turn, modulate autocrine and paracrine signaling mechanisms in the TME. For example, E-cadherin adhesion proteins were found to be upregulated in 12 individual spheroid cultures made from each H&N cancer cell line (FaDu, HLaC78, Hep-2, Hep-2-Tax, HLaC79, HLaC79-Tax, HPaC79, HSmC78, CAL-27, PE/CA-PJ41, SCC4, HNO210) but not in any of the corresponding 2D monolayer controls (32). As such, 3D sphere-like culture models, as of spheroids, are essential to emulate the physical and biochemical characteristics of the solid tumor shape in the evaluation of cell–cell/–ECM crosstalk and pharmacokinetics of cancer drugs (32, 33).

## Heterogeneous cell types

Recently, multicellular *in vitro* models have been developed for lung (37), breast (38), and pancreatic (39) cancer research. Such model is particularly useful to study the crosstalk between cells in response to cancer drugs. For example, a triple coculture pancreatic model was developed to create a hetero-, multicellular tumor spheroid consisting of pancreatic cancer cells, fibroblasts, and endothelial cells for the investigation of the TME response to chemotherapy (39). To mimic the heterogenous TME in H&N cancer, cell lines such as CAL-27, CAL-33, Detroit 562, Hep2, Hep3, FaDu, SCC-4, UM-SCC-3, UM-SCC-4, and UM-SCC-17A, among others, are widely used in *in vitro* 2D and 3D H&N cancer models (40).

Being able to model a heterogeneous cell population *in vitro* is key to understand the complex interactions of cancer, stromal, and immune cells, and their collective response to the testing drugs within the TME (**Figures 2C**, **D**). For instance, a cisplatin sensitivity study used a simple 2D transwell system with Boyen’s chambers to coculture patient-derived CAFs and pharyngeal cancer cell lines (FaDu and Detroit 562) (41). Clonogenic survival and gene inspection showed that CAFs notably affected the colony-forming and cisplatin-sensitizing capabilities of pharyngeal cancer cells through the paracrine signaling of VEGFA, PGE2S, COX2, EGFR, and NANOG. As 2D transwell systems can incorporate two cell types at most, enhancing the complexity of *in vitro* models is a necessary step to better mimic the 3D tumor cell heterogeneity in H&N and other tumors. However, one major challenge of multicellular coculture models is the cross-contamination of culture media (42). To address this challenge, microfluidic platforms can be used to compartmentalize heterogeneous cell populations within the same culture platform (43, 44). One plausible strategy is to culture individual cell populations in separate compartments sharing a constantly irrigated channel with cultured media. The shared media will then contain paracrine factor secretion aiding the multicellular interactions of the individual cellular compartments.

## Hypoxic environment and fenestrated vasculature

Tumor hypoxia is a notable factor of avascular solid tumor cores and micrometastases in cancer development (45). The TME of H&N cancer may have regions with oxygen levels as low as <5 mmHg at hypoxic sites (46). Fenestrated vasculature in hypoxic niches leads to vessel leakage, which limits an effective supply of oxygen, nutrients, and therapeutics to the tumor core. Hypoxic cues, namely, oxygen deprivation and irregular irrigation, are thus two key parameters to be considered in the design of effective *in vitro* H&N cancer models (**Figures 2E**, **F**).

Regarding oxygen deprivation, hypoxic gradients can be created by utilizing 3D *in vitro* culture geometry (47) or hypoxic culture chambers with microfluidics (48). For instance, spheroid cultures have been created to generate three geometrical regions with distinctive hypoxic gradients, namely, (I) an outer high-oxygen/nutrient-proliferative region, (II) a middle medium-oxygen/nutrient senescence region, and (III) a low-oxygen/nutrient necrotic region found in the spheroid core (36, 45).

Concerning irregular irrigation, static cultures do not translate the capillary supply as of *in vivo* systems (49). To this end, microfluidic technologies hold great promises to mimic the irregular blood supply of tumors by precisely controlling and monitoring the flow rate of media (ranging in microliters per minute) with integrated microchannels and a sensing element into the culturing platform (50). Hypoxic profiles can also be tuned by integrating spheroid models into microfluidic platforms. The cellular uptake of chemotherapy drugs can then be imaged along specific hypoxic gradients with real-time microscopy (51).

## Advanced *in vitro* models for head and neck cancer drug screening and evaluation

**Figure 3:**
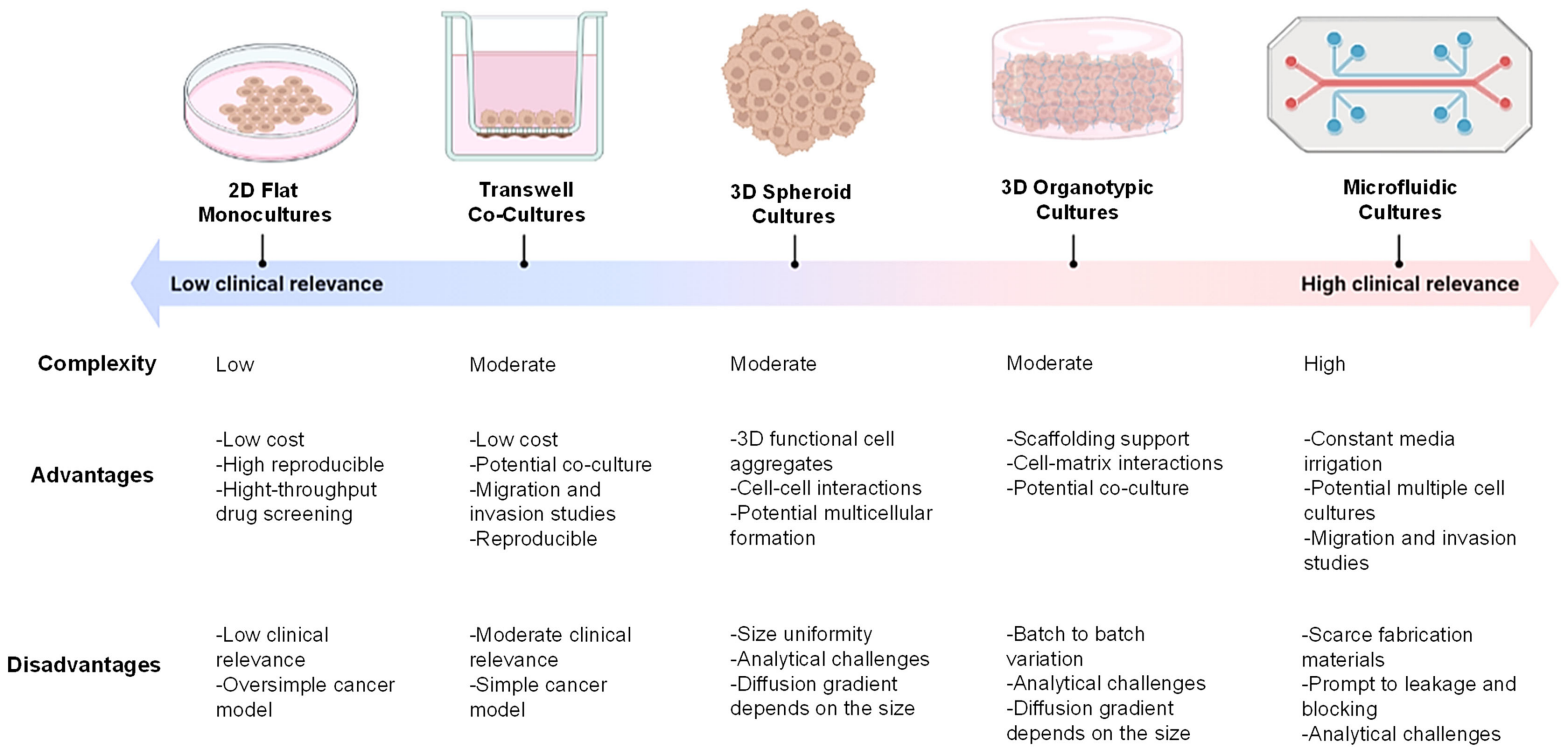
Common *in vitro* cancer models. Two-dimensional (2D) flat monolayer cell cultures grown on plastic or glass surfaces. Transwell systems with Boyden’s chamber inserts for cellular cocultures. 3D spheroid-based systems by forced aggregation of cells into a 3D construct. 3D organotypic systems by culturing cells within a matrix such as a hydrogel. Microfluidic-based culture systems by culturing cells within a microchannel with fluid circulation. Figure created with BioRender.com.

The most common evaluation platform for drug development in H&N cancer is conventional 2D *in vitro* models thus far due to their low cost, high reproducibility, and potential coculture capability (52). However, 2D *in vitro* models are unable to (I) mimic the physical geometry of tumor, (II) avoid the cross-contamination of culture media in multicellular models, and (III) mimic the oxygen deprivation and irregular irrigation of the hypoxia region, which are key factors in the evaluation of tumor progression, chemoresistance, and treatment response (35, 36, 52). Advanced *in vitro* systems, including spheroids, 3D scaffolds, and microfluidic devices, have thus been developed to overcome these barriers (53). Although the application of these culture platforms to model H&N cancer microenvironment and its drug discovery is still in its infancy, recent research on H&N cancer has been using 3D *in vitro* models to advance the growing need of these systems for clinical translation (**Figure 3**).

## Spheroid models

**Figure 4:**
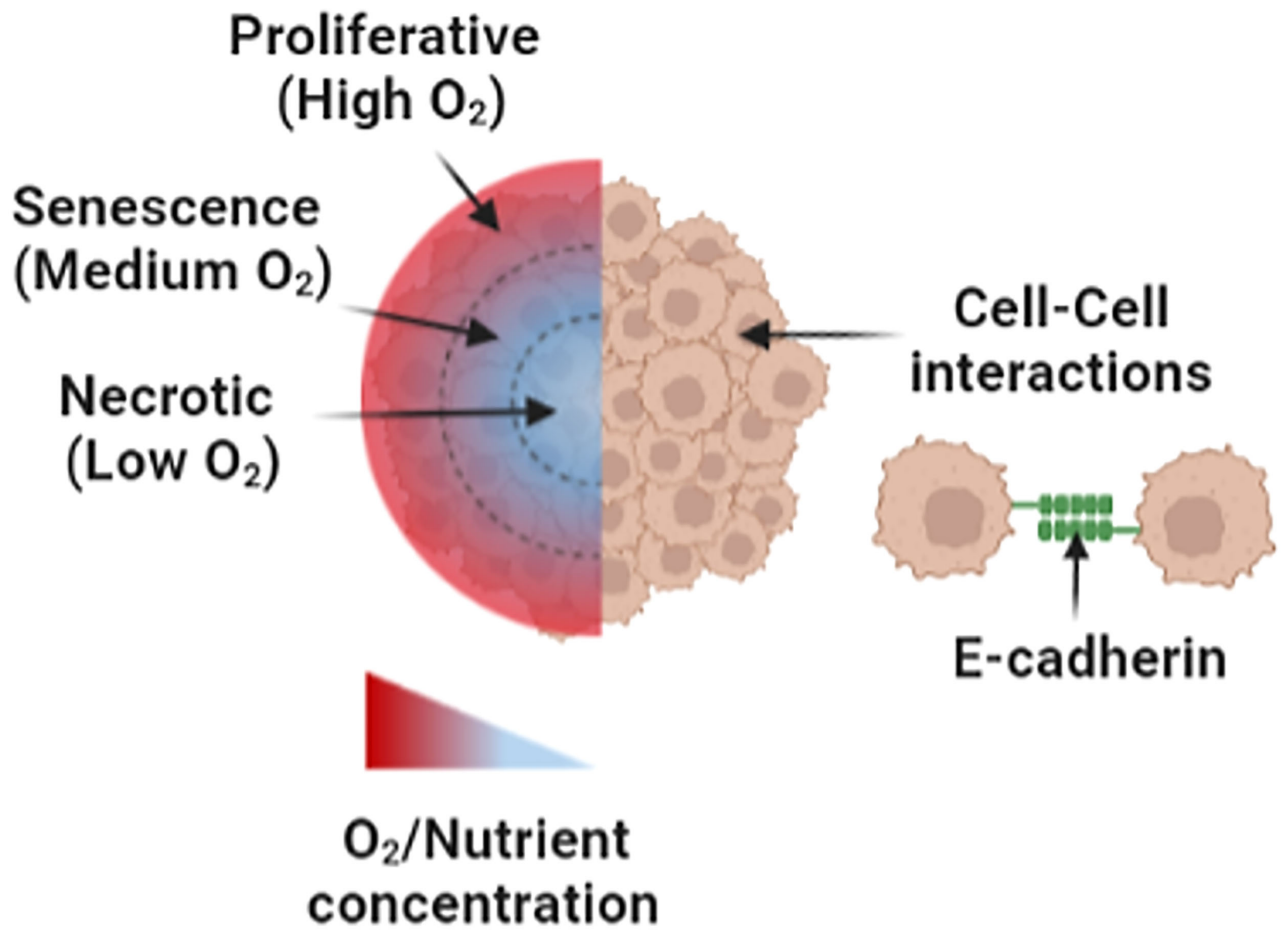
An illustration of spheroid culture model. Hypoxic gradients within spheroid cultures comprise an outer high-oxygen/nutrient region, a middle medium-oxygen/nutrient region, and a low-oxygen/nutrient region. In addition, cell–cell interactions take place in the spheroid model *via* functional cell aggregation and E-cadherin binding. Figure created with BioRender.com.

Spheroids are functional aggregations of cells that are generally formed *via* forced floating aggregation, hanging drop, or organotypic hydrogel embedment methods (52). The forced floating aggregation methods are most commonly used in H&N cancer models (32–34, 54–57) (**Table 1**, **Figure 4**). The forced floating method is to use low-attachment well-plates that hinder the cell–substrate interaction and promote cell self-aggregation. In addition, hanging drop and hydrogel embedment methods were also used to fabricate H&N cancer spheroids. The hanging drop methodology is to place a drop of cell suspension on the underside of culture plates that cells can aggregate and form spheroids at the drop tip (58, 59). For the organotypic hydrogel embedment approach, cell suspensions are pipetted into an ECM-based hydrogel for cellular support, self-assembly, and spheroid formation (60, 61).

**Table 1:** Spheroid models in head and neck (H&N) cancer research.

Author	Aim	Drug Stimulant	Culture Model Design and Components				Analytic Outputs	Main Findings
			Single vs. Multicellular Cultures	Primary vs. Cell Lines	2D vs. 3D Geometry	Hypoxic Cues		
Schmidt et al. (32)	To compare the effect of 2D and 3D culture methods regarding gene expression in terms of cell junctions, cell adhesion, cell cycle, and metabolism	NS	Single	**Primary:** NS **Cell lines:** - FaDu -HLaC78 -Hep-2 -Hep-2-Tax -HLaC79 -HLaC79-Tax -HPaC79 -HSmC78 -CAL-27 -PE/CA-PJ41 -SCC4 -HNO210	**2D:** Monolayer control **3D:** Forced floating method	NS	-RNA extraction -RNA quality control -Microarray analysis -Real-time PCR -Scanning electron microscopy	-Spheroid tight formation was dependent on the upregulation of E-cadherin (cell adhesion) and downregulation of Ki67 (cell proliferation) in comparison to monolayer controls
Melissaridou et al. (33)	To compare the effect of 2D and 3D culture methods on cell proliferation, response to anticancer drugs, and EMT profiles	-Cetuximab -Cisplatin	Single	**Primary:** NS **Cell lines:** -LK0858B -LK0902 -LK0917 -LK1108 -LK1122	**2D:** Monolayer control **3D:** Forced floating method	NS	-Clonogenic assay -Tunel staining -CellTiter 96^®^ Proliferation Assay -Western blotting -RT-qPCR	-Spheroids presented a cancer stem cell-like phenotype (upregulation of EMT-associated proteins). -Drug effects were significantly different on spheroids compared to monolayer control.
Azharuddin et al. (34)	To compare the effect of 2D and 3D culture methods regarding chemoresistance	-Cisplatin -Doxorubicin -Methotrexate	Tri-culture (cancer cells)	**Primary:** NS **Cell lines:** -LK0902 -LK0917 -LK1108	**2D:** Monolayer control **3D:** Forced floating method	NS	-CellTiter 96^®^ Proliferation Assay -Live-cell imaging calcein-AM -Ros DCFDA assay -Flow cytometry	-Drug vulnerability and potential chemoresistance was predicted by analyzing efflux pump (ABC pump) activities. -Comparative response of multidrug resistance, drug efflux capability, and reactive oxygen species on treated cells.
Essid et al. (48)	To compare the effect of 2D and 3D culture methods on EMT, cancer stem cell, and hypoxia markers	Hypoxia 1% O_2_ chamber (monolayer)	Single	**Primary:** NS **Cell lines:** -CAL-33	**2D:** Monolayer control **3D:** Forced floating method	✓	-Clonogenic assay -Western blotting -Immunofluorescence staining -RT-PCR	-Serum in media was reported to revert EMT, cancer stem cell, and hypoxia phenotype. -Spheroids cultured under hypoxia (1% O_2_) showed increased carbonic anhydrase IX, vimentin, N-cadherin, glioma-associated oncogene homolog 1, and decreased E-cadherin.
Basheer et al. (47)	To compare the effect of hypoxic and normoxic culture methods on HIF-1α–CCR7 correlation	Hypoxia, low O_2_ or CoCl_2_ to cell culture medium	Multicellular	**Primary:** NS **Cell lines:** -OSC-19 -FaDu -SCC-4 -A-253 -Detroit-562	**2D:** Monolayer control **3D:** Spheroid formation Not specified	✓	-Immunofluorescence staining -Immunoblotting -Flow cytometry	-HIF-1α expression (hypoxia) was associated with the expression of CCR7 (migration marker). -Correlation between HIF-1 α and CCR7 was noted in early histological xenograft cancer samples
Hagemann et al. (54)	To compare 2D and 3D methods as chemotherapy and radiotherapy testing platforms	-Cisplatin -5-FU -2-Gy radiation	Single	**Primary:** -Tumor biopsy from H&N squamous cell carcinoma **Cell lines:** -CAL-27 -FaDu -PiCa	**2D:** Monolayer control **3D:** -Forced floating and -Hanging drop methods	NS	-WST-8 assay -ELISA	-Forced floating method was reported to be safer and more reliable than the hanging drop method. -Proof-of-concept data concerning spheroids as a therapy screening platform. -Spheroid growth was reduced after chemoradiation treatment. Significant negative impact was noted with the cisplatin + radiation treatment compared to cisplatin alone.
Goričan et al. (55)	To evaluate a 3D model as a therapy testing platform	All-trans retinoic acid (ATRA)	Single	**Primary:** NS **Cell lines:** -FaDu	**2D:** NS **3D:** Forced floating method	NS	-Immunofluorescence staining -qPCR -Flow cytometry -Western blotting -HTS	-A new cancer stem cell–enriched spheroid model adaptable for HTS of anticancer stem cell compounds -ATRA treatment was reported to reduce cancer stem cell markers.
Magan et al. (56)	To evaluate a 3D model as chemotherapy and immunotherapy testing platforms	-Cisplatin -Cetuximab	Two-culture	**Primary:** Patient-derived cancer-associated fibroblasts **Cell lines:** -LK0902 -LK0917 -LK1108	**2D:** NS **3D:** Forced floating method		-Immunofluorescence staining -TUNEL assay -RT-qPCR - CellTiter 96^®^ Proliferation Assay	-Cancer-associated fibroblasts increased cancer cell proliferation and EGFR expression in cocultured tumor spheroid -EGFR-overexpressed spheroids showed increased response toward cetuximab after 72-h exposure -Ki67 overexpression was noted in tumor cells treated with cisplatin for 72 h
Kochanek et al. (57)	To evaluate a 3D model as a chemotherapy testing platform	-Doxorubicin	Single	**Primary:** NS **Cell lines:** -FaDu -CAL-27 -CAL-33 -OSC-19 -Detroit-562 -BIRC-56 -PCI-13 -PCI-52 -UM-SCC-1 UM-22B -SCC-9 -HET-1A	**2D:** NS **3D:** Forced floating method	NS	-Immunofluorescence staining -Widefield microscopy -LIVE/DEAD staining -Proliferation assay -Mitochondrial mass and membrane potential assay	-Cells at the outer layer of the spheroid showed higher drug uptake compared to cores after 1-day exposure -Spheroid morphology was altered after 1-day drug exposure

NS, not studied.

With the introduction of spheroid H&N models, researchers were able to better decipher the epithelial–mesenchymal transition (EMT) mechanism under a hypoxic environment with or without cancer drugs (62). For example, Melissaridou et al. (33) compared 2D and 3D cell cultures from five H&N squamous cell carcinoma–derived cell lines in their expression of EMT and stemness markers as well as response to cetuximab and cisplatin drugs. EMT-associated and stem cell markers including CDH1, NANOG, and SOX2 were upregulated in 3D spheroid groups but not in 2D monolayer controls. In addition, the spheroid groups showed increased resistance to cisplatin and cetuximab treatments compared to 2D monolayer cultures. Essid et al. (48) developed spheroids from a human tongue cell line to investigate the relationship between EMT and hypoxia. These spheroids were grown in hypoxic chambers subjected to 1% O_2_ for 30 days. Results showed an increased mRNA expression in E-cadherin and N-cadherin as well as carbonic anhydrase 9, a hypoxic marker, in the spheroid hypoxic cores.

To further investigate the effect of hypoxia on the treatment response in H&N cancers (**Figure 4**), Basheer et al. (47) analyzed protein expression on five H&N cancer cell lines under normoxia and hypoxia in both OSC-19 spheroid cultures and monolayer controls using Western blot, flow cytometry, and immunofluorescence staining. The protein expression of CCR7, a chemokine receptor associated with hypoxia, was found significantly higher in the hypoxic core of the spheroid cultures compared to monolayer and normoxic controls. All in all, previously mentioned results pointed to the importance of tumor-like geometries as presented in spheroid models for the evaluation of drug sensitivity and cytotoxicity.

## Future prospects

New 3D bioprinting techniques such as inkjet-based, pressure-assisted, and laser-assisted approaches (63) hold new promises for fabricating complex organotypic tumor spheroids in terms of cellularity and architecture (64). To fabricate multicellular spheroids, bioprinting allows the layer-by-layer precise assembly of 3D biological constructs. Synthetic polymers (e.g., polycaprolactone) and naturally derived polymers (e.g., alginate) are commonly used as bioinks to resemble the tissue-specific ECM (65, 66). Bioinks can also be printed with multiple cell types (squamous cell carcinomas, CAFs, and pro-tumor macrophages of H&N tumors) by using pressure-assisted and laser-assisted printing approaches (63). The incorporation of cancer stem cells may further mirror the aggressive H&N TME (55, 67) in the bioprinted construct due to the self-renewal and differentiation capabilities of these cell types. In addition, physiological cues such as 3D tumor geometry, cell heterogeneity, and normoxic-to-hypoxic strata can thus be recreated to induce cel–cell/–ECM interactions as expected in the H&N TME (68).

Further, a multi- and heterogeneous-layer geometry of the tumor spheroids can be bioprinted by implementing cell-laden bioink deposition with zone-specific techniques, for example, by varying pore-size and interconnectivity (63, 66, 69). As a result, each layer of the organotypic spheroid can have individual TME cell populations and ECM compositions to better mimic hypoxic niches within the tumor-like *in vitro* models (63). Within the 3D organotypic models, organoids that are specific 3D cell–embedded models consisting of stem or patient-specific cells and ECM constituents in the form of a multilayer geometry are very desirable H&N TME models (70). The future perspective of organoids is further discussed in the Future Outlook section.

## Organotypic models

**Figure 5:**
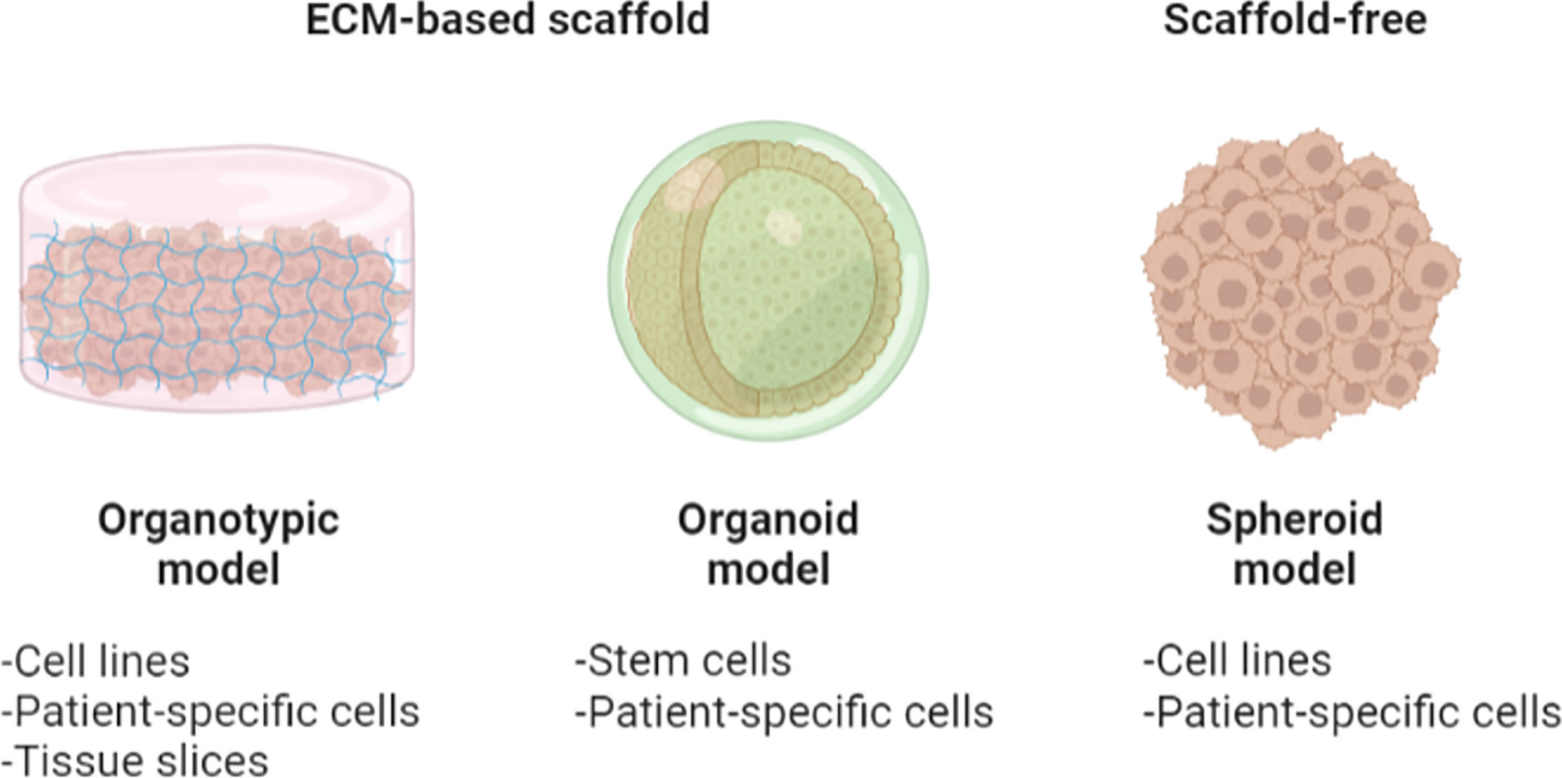
An illustration of organotypic culture models. Organotypic models provide cell–cell/ECM interactions within the culture model. Organotypic models are 3D *in vitro* platforms comprising the embedment of disaggregated cells/tissues in ECM-based scaffolds. Particularly, organoids are those organotypic models derived specifically from stem or patient-specific cells. Spheroids may be fabricated using one or multiple conventional cell lines or patient-derived cells, with or without the use of ECM-based embedment. Figure created with BioRender.com.

Organotypic models provide intracellular communication between cells embedded in ECM-based scaffolds (71–74) (**Figure 5**). A 3D scaffold-based *in vitro* model aims at recapitulating the native tissue’s ECM microenvironment in terms of mechanical stability and structural architecture in the support of cell signaling, migration, survival, and growth (75). The materials used to make biological scaffolds are mostly obtained from natural or synthetic polymers, often in aqueous form. To convert the aqueous materials to a gel-like scaffold, crosslinking methods such as UV radiation, enzymatic reactions, and temperature changes have been adopted for sol–gel transitions in most *in vitro* cancer model developments (76).

To date, organotypic H&N models comprise the use of patient-derived H&N squamous cells together with decellularized extracellular matrix (dECM) (77–80) or synthetic ECM substitutes (60, 61, 81–83) as the most common constituent materials (**Table 2**). In particular, dECM scaffolds are often selected for cancer modeling, owing to their retained bioactive molecules (e.g., collagen, proteoglycans, and glycoproteins) (75) to support H&N cancer and TME cells for organoid formation. In addition, synthetic ECM substitutes such as the commercially available Matrigel^®^, which is derived from mice sarcoma (84), are also used for fabricating organotypic H&N cancer models (60, 77, 80). However, Matrigel^®^ is reported with single-batch variations that cause a significant concern on mechanical inconsistency, especially in fabricating reproducible organoids even when using the same batch of the product (84).

**Table 2:** Organotypic models in H&N cancer research.

Author	Aim	Drug Stimulant	Culture Model Design and Components				Analytic Outputs	Main Findings
			Single vs. Multicellular Cultures	Primary vs. Cell Lines	2D vs. 3D Geometry	Hypoxic Cues		
Tanaka et al. (60)	To compare 2D vs. 3D methods as a chemotherapy sensitivity platform	-Cisplatin -Docetaxel	Single	**Primary:** -Tumor biopsy from H&N squamous cell carcinoma **Cell lines:** -MDA-HN2016-2 -MDA-HN2016-18 -MDA-HN2016-21	**2D:** Monolayer control **3D:** Forced floating method and then transferred into Matrigel^®^	NS	-DNA extraction -STR profiling -Western blotting -Clonogenic assay	-Patient-derived organotypic models were useful as testing platforms for chemotherapy agents. -Seven 2D cell lines and 13 organoid cell lines produced after this study -Obtained cell lines presented chemoresistance cues as a tissue source
Driehuis et al. (61)	To compare 2D vs. 3D methods as a photodynamic therapy testing platform	Photosensitizer (binds EGFR) for photodynamic therapy	Single	**Primary:** -Tumor biopsy from H&N squamous cell carcinoma **Cell lines:** - UM-SCC-14C -CRL-1555 -human cervical carcinoma cell line HeLa (CCL-2) -human embryonal kidney cell line HEK293T (CRL-3216)	**2D:** Monolayer control **3D:** Basement Membrane Extract type 2 (an ECM mimetic agent) in media	NS	-qPCR -Flow cytometry -Immunofluorescence staining -PDT assay	-Patient-derived organotypic model had similar EGFR expression as a tissue source. -These models were useful as testing platforms for EGFR-targeted therapy.
Zhao et al. (77)	To compare 2D vs. 3D methods as chemotherapy screening and a regenerative platform	Cisplatin	Single	**Primary:** -Patient-derived tongue squamous cell carcinoma and cancer-associated fibroblasts **Cell lines:** -CAL-27	**2D:** Monolayer control 3D: Decellularized tongue extracellular matrix From mice, pig, and rat collagen I/Matrigel^®^ matrix	NS	-Immunohistochemistry and immunofluorescence staining -Scanning electron microscopy -Transmission electron microscopy -Atomic force microscopy -DNA quantification -Proteomic analysis -MTT assay -Scratch assay	-3D scaffold derived from tongue squamous cell carcinoma as *in vitro* culture support and migration -3D ECM-like platform for drug testing -Mouse-derived dECM scaffold showed increased cell adhesion, survival, and differentiation compared to control -Cisplatin exposure data showed the heterogeneity of cisplatin response within the muscle and basal layers of the mouse-derived dECM scaffold *via* cell cytotoxicity and caspase 8 positive staining compared to monolayer control.
Burghartz et al. (78)	To compare 2D vs. 3D methods as *in vitro* support model	NS	Single	**Primary:** Human salivary gland epithelial cells **Cell lines:** -CAL-27	**2D:** Monolayer control **3D:** Decellularized porcine jejunum matrix	NS	-Immunofluorescence staining -Scanning electron microscopy -Transmission electron microscopy -Amylase Assay Kit -RT-PCR	-3D ECM-like platform for potential radiotherapy use -Gene expression of α-amylase was higher in 3D mono- and coculture compared to 2D monoculture
Ayuso et al. (79)	To compare 2D vs. 3D methods as dual drug- screening platform	-AZD8055 (mTOR inhibitor) -Cetuximab (Erbitux)	Two-culture	**Primary:** -Patient-derived cancer-associated fibroblasts **Cell lines:** -UM-SCC-1 -UM-SCC-47	**2D:** Monolayer control **3D:** Spheroid hanging drop (cultured without fibroblasts) 3D collagen hydrogel (cultured without fibroblasts)	NS	- CellTiter 96^®^ Proliferation Assay -Immunofluorescence staining	-3D ECM-like platform as coculture setup for drug testing and EGFR pathway analysis -Cell cytotoxicity data showed higher drug resistance response in the coculture (1.4-fold increase) and 3D culture groups (2.6-fold increase) compared to 2D monocultures
Tuomainen et al. (80)	To compare 2D vs. 3D methods as a drug- screening platform	-EGFR (gefitinib, erlotinib, cetuximab, canertinib, and afatinib) -MEK (trametinib, TAK-733, selumetinib, refametinib, pimasertib, and binimetinib) -mTOR (temsirolimus, sirolimus, ridaforolimus, PF-04691502, omipalisib, everolimus, dactolisib, and apitolisib)	Single	**Primary:** NS **Cell lines:** -UT-SCC-8 -UT-SCC-14 -UT-SCC-24A -UT-SCC-24B -UT-SCC-28 -UT-SCC-42A -UT-SCC-42B -UT-SCC-40 -UT-SCC-44 -UT-SCC-73 -UT-SCC-81 -T-SCC-106A	**2D:** Monolayer control **3D:** Matrigel^®^ and a leiomyoma-derived matrix “Myogel”	NS	-Drug sensitivity and resistance testing - CellTiter 96^®^ Proliferation Assay -Meta-analysis of Clinical Data -Immunoblot analysis	-3D ECM-like platform for drug testing and pathway analyses -Cells seeded in Myogels showed significantly lower EGFR and MEK inhibition activity -Cells seeded in both scaffolds showed a low mTOR inhibition activity in most of the cell lines
Young et al. (81)	To compare 2D vs. 3D methods as radiotherapy- screening platform	5 or 10 Gray	Two-culture	**Primary:** -Patient-derived Cancer-associated fibroblasts **Cell lines:** -CAL-27	**2D:** Monolayer control **3D:** Tissue Roll for the Analysis of Cellular Environment and Response (TRACER) construct a collagen gel and cellulose scaffold	✓	-MTT assay -Immunofluorescence staining -Hypoxia (EF5) staining -Live/Dead staining -Cell migration -Clonogenic assay	-3D ECM-like platform as coculture setup for radiotherapy and hypoxia analysis -Increased cell migration and invasion of tumor-stroma cocultures within the layers of the tissue roll construct -No significant radiation resistance of tumor-stroma cocultures within the layers of the tissue roll construct
Lee et al. (82)	To compare 2D vs 3D methods as chemotherapy testing platform	-Cisplatin -Docetaxel	Two-culture	**Primary:** -Tumor biopsy/explants from H&N squamous cell carcinoma **Cell lines:** -NS	**2D:** Monolayer control **3D:** Dissociated epithelial cells seeded on a mixture of solidified fibrin glue and tumor explants	✓	-Cell counting kit-8 (CCK-8) -LIVE/DEAD assay using -LOX-1 a hypoxia probe	-Tumor explants were reported to present hypoxic cues, and drug screening sensitivity -Tumor explants in fibrin matrix survived over 10 days while those explants without the matrix survived less than 8 days
Engelmann et al. (83)	To compare HPV-associated organotypic explants as radiotherapy testing platform	2 Gray	Multicellular	**Primary:** -Tumor biopsy/explants from H&N squamous cell carcinoma **Cell lines:** -NS	**2D:** NS **3D:** Dermal equivalents from viscose fiber fabric embedded with fibroblast for ECM production H&N squamous cell carcinoma as tissue slices	NS	-H&E staining -Immunohistochemical staining -Immunofluorescence staining -PCR -Motility and invasiveness analysis -Cell viability, proliferation, and apoptosis assays	-3D ECM-like platform for radiotherapy use -Radioresistant tumor cells and morphological variations were noted after 5-day fractionated irradiation exposure -Tumor slices/explants in dermal equivalents remained viable for up to 21-day cultures

NS, not studied.

In an effort of developing patient-specific organotypic models, Tanaka et al. (60) combined an epithelial cell sheet, the Matrigel^®^, and individual squamous cell carcinomas derived from 43 biopsies of H&N cancer patients. The organotypic models were subjected to the exposure of cisplatin and docetaxel for eight consecutive days (60). Results showed that these models displayed a patient-specific chemoresistant response. For example, the MDA-HN-2C organoid group developed resistance to cisplatin and docetaxel, corresponding to that of the individual patient donor with recurrent H&N cancer. In addition, the organoid-like models showed increased resistance to both drugs in comparison to that of 2D monolayer controls. The proposed patient-derived organoid (PDO) platform served a notable step toward the application of predicting patient-specific H&N drug sensitivity *in vitro*.

One advancement of the cancer organotypic model is to approximate the heterogeneity of tissue strata as seen in the tumor architecture. For instance, in H&N tumor, tissue strata mostly comprise squamous epithelia, basal strata, stroma, and lamina propria. Zhao et al. (77) investigated whether the tissue sources of dECM would result in a specific stratum architecture of the scaffold that might, in turn, affect the drug response of cancer cells. Mouse, rat, and pig tongue tissue samples were decellularized and used to fabricate scaffolds with patient-specific cancer-associated fibroblasts and CAL-27 cells. Hematoxylin & eosin staining, scanning electron microscopy, and transmission electron microscopy showed a similar histological stratum architecture of the three dECM scaffolds. Further investigation using a mouse dECM scaffold showed that the elastic modulus of mouse dECM scaffolds was comparable to that of native mouse tongue tissue (0.503 MPa vs. 0.567 MPa). Compared to monolayer non-scaffold controls, mouse-derived dECM scaffolds showed improved cell adhesion, proliferation, and survival after 14 and 28 days of cultures in the absence of drug exposure. After a 2-day exposure of cisplatin, an apoptotic marker, namely, caspase 8, showed distinctive staining patterns across the strata of mouse-derived dECM scaffolds. For instance, cancer cells at the muscle fiber layer of the scaffold expressed stronger caspase 8 expression than those at the basal layer of the scaffold, possibly owing to the drug-penetration gradients.

Aside from the evaluation of dECM sources, Ayuso et al. (79) compared 3 culture models, namely, (I) 2D monolayer cocultures with primary cancer-associated fibroblasts and H&N cancer cell lines (UM-SCC-1 and UM-SCC-47), (II) 3D collagen hydrogel scaffolds seeded with H&N cancer cells, and (III) 3D H&N cancer cell spheroids of their responses to cetuximab and an mTOR inhibitor. Cell cytotoxicity results indicated a stronger drug resistance response in the coculture (1.4-fold increase) and 3D culture groups (2.6-fold increase) compared to 2D monocultures. No statistical comparison was reported between the two 3D culture groups. Nevertheless, the differentiated drug resistance between the 2D and the 3D culture groups may be associated with the geometry-induced drug impediment.

High-throughput screening (HTS) with organotypic models is one critical advancement of scaffold models for immune-oncology and drug discovery (85). Using 384-well plates, Tuomainen et al. (80) evaluated the effect of 19 immunotherapy drugs on 12 H&N cancer cell lines seeded within 3D scaffolds inserted in those plates. The 19 immuno-drugs were inhibitors of 5 EGFR (gefitinib, erlotinib, cetuximab/erbitux, canertinib, and afatinib), 6 MEK (trametinib, TAK-733, selumetinib, refametinib, pimasertib, and binimetinib), and 8 mTOR (temsirolimus, sirolimus, ridaforolimus, PF-04691502, omipalisib, everolimus, dactolisib, and apitolisib). The testing scaffolds included Matrigel^®^ and human-derived leiomyoma referred to as Myogel. Compared to Matrigel^®^, cells embedded in Myogels showed significantly lower EGFR and MEK inhibition activity after 72 h of drug inspection. Normalized HTS drug response profiles consisted of four activity levels based on a drug-sensitivity score (DSS) and artificial cutoff points: inactive DSS < 5, low 5 ≥ DSS < 10, moderate 10 ≥ DSS < 15, and high DSS ≥ 15 (80). Overall, a low activity of mTOR inhibitors was consistently found in most of the cell lines from both Matrigel^®^ and Myogel scaffold models. Results from this study provided early evidence of the reliability and predictability of using HTS organoid platforms in the evaluation of cancer therapeutics.

In addition to chemotherapy drug–related studies, Young et al. (81) developed a 3D tissue construct of a collagen and cellulose tissue roll scaffold “TRACER” for radiation therapy screening. The FaDu cell line and primary cancer–associated fibroblasts, stromal cells, were transfected with green fluorescent protein and mCherry, respectively. Both cells were seeded into the cellulose layer (cancer-associated fibroblasts in layer 1 and FaDu in layer 3) with or without a central collagen/agarose layer to separate the coculture. The cell-seeded TRACER was rolled onto an acrylic core placed into custom-made 50-ml Falcon tubes and then subjected to 5- or 10-Gray radial arc radiations. Clonogenic results indicated that no radioprotective behavior from the CAFs was observed in the cocultures regardless of the presence of the central layer after 24-h culture. In a separate study, x-ray radiation (0–15 Gray) was found to downregulate HeLa cancer cell proliferation, cell viability, vinculin, and α-tubulin expression in 2% agarose hydrogels with 250 µm of diameter compared to 2D flat counterparts (86). Although results from these two radiation studies were not fully corroborated, 3D tissue constructs with cocultures showed the potentials of elucidating epithelial–stromal interactions of tumor response to radiation exposure.

## Future prospects

Organotypic models have demonstrated great possibilities for approximating the TME and supporting the HTS cancer drug platform. Several technical challenges remain to adapt the organotypic models to fulfill the two aforesaid promises. Organotypic fabrication is complex, especially considering the scaffold embedment that influences the therapeutic response based on the scaffold’s composition and network (87). For instance, these models have not been fully designed to incorporate the irrigation features of tumor modeling. One possibility is to place the scaffolds into microfluidic channels to recapitulate the constant irrigation features of native tumor or healthy tissues with bioprinting and electrospinning techniques (88). Electrolyte-assisted electrospinning can further help to fabricate nanofibrous membranes through electrostatic forces to draw charged threads of dissolved polymers to a grounded electrolyte solution (89). These nanofiber membranes can be located inside microfluidic channels for tissue-engineered scaffolds (89). By integrating electrospinning and microfluidic technologies, scaffold-based models can better meet the functionality of continuous monitoring and irrigation of cancer therapeutics.

## Microfluidic platforms

**Figure 6:**
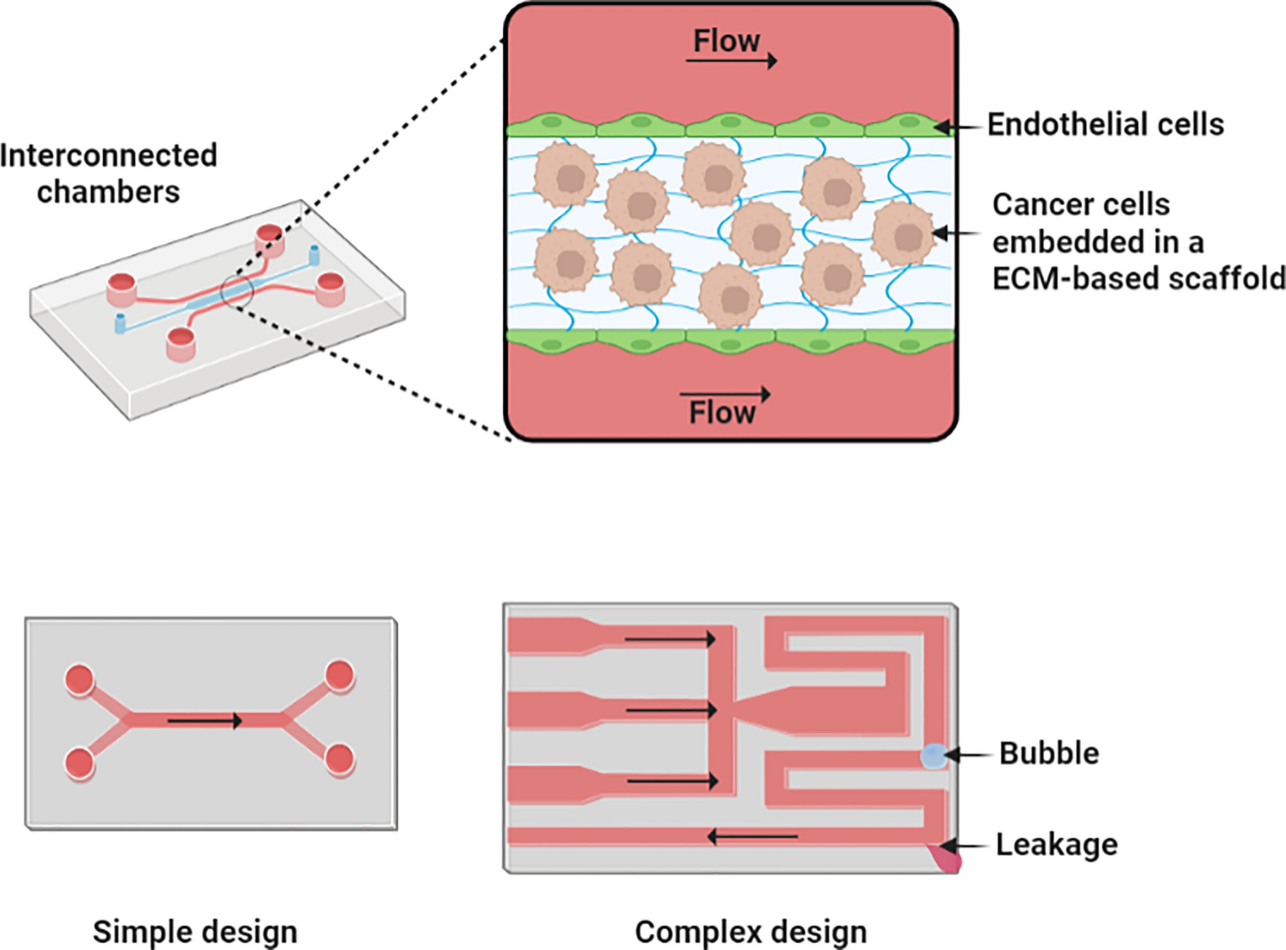
An illustration of microfluidic culture models. Microfluidic devices comprise the interconnection of chambers and grooves sharing low volumes of liquids. A more complex design with more channels and chambers can enhance its physiological representation but may also increase the chance of challenges as bubble blocking and liquid leakage. Figure created with BioRender.com.

Microfluidic platforms are micromanufactured devices with interconnected chambers, membranes, and grooves that share low volumes of fluids (**Figure 6**), which have been widely applied for *in vitro* modeling such as organ-on-a-chip models (49, 90–95) and point-of-care systems (96). In cancer research, microfluidic platforms are mostly fabricated using lithography and surface micromatching techniques with polydimethylsiloxane, silicon, glass, polycarbonate, and polymethylmethacrylate as main materials (49, 91–93, 97–99). Flow mechanisms can be implemented through a passive or an active approach within the microfluidic device. Passive flow can be driven by gravity, hydrostatic pressure, surface tension, or osmotic pumps (93). Active flow mechanism, which is commonly used in H&N microfluidic devices, involves the use of peristaltic (2 µl/min to 10 L/min), syringes (0.012 nl/min to 0.3 L/min), and pressure-driven pumps (nl/min to ml/min) (49, 91, 93, 96–100).

Microfluidic platforms support simultaneous compartmentalization of multiple cancer cell populations with constant culture media irrigation (90). This compartmentalization with dynamic flow features allows for the programmatic control and real-time monitoring of cancer cell–vasculature interplay through the interconnected cellular compartments of the platform (49, 91, 101). Most chemotherapy drugs are also delivered intravenously that flow dynamically through blood vessels to the tumor vasculature and extravascular tissues (102). The dynamic flow feature of microfluidic devices can thus resemble the transportation of intravenous systemic treatment and help to evaluate its pharmacokinetics in a more precise, controllable manner. Chemotherapy drugs, such as paclitaxel, cisplatin, and 5-fluorouracil, have been tested with microfluidic devices in H&N cancer research (**Table 3**).

**Table 3:** Microfluidic Devices in H&N Cancer Research.

Author	Aim	Drug Stimulant	Culture Model Design and Components				Analytic Outputs	Main Findings
			Single vs. Multicellular Cultures	Primary vs. Cell Lines	2D vs. 3D Geometry	Hypoxic Cues		
Hattersley et al. (49)	A dynamic culture method as chemotherapy screening platform	-5-FU -Cisplatin	Single	**Primary:** Patient-derived H&N squamous cell carcinoma **Cell lines:** -NS	**2D:** Unexposed control Dynamic flow **3D:** Multimicro channels Dynamic flow Syringe pump	NS	-H&E staining -Lactose dehydrogenase release -WST-1 metabolism -Trypan blue -Cytochrome C analysis	-Preclinical model for personalized medicine and testing -H&E staining showed the retention of multilayer tissue strata -Combination therapy presented higher levels of cytochrome C compared to untreated control
Riley et al. (91)	A dynamic culture method as drug screening platform	-Etoposide (topoisomerase II inhibitor) -SP600125 (JNK inhibitor)	Single	**Primary:** Human thyroid tissue samples **Cell lines:** -NS	**2D:** Unexposed control Dynamic flow **3D:** Tissue chamber Dynamic flow Syringe pump	NS	-Hematoxylin and eosin -Flow cytometry -Trypan blue -Immunohistochemistry staining -Functional analysis - Lactose dehydrogenase release -TUNEL assay -Immunoblot analysis	-Preclinical model for personalized medicine and testing -H&E staining showed the retention of multilayer tissue strata -Thyroid biopsies were considered functional due to the production of T4 during the culture period -Increased apoptosis on thyroid samples after the perfusion of both drugs in comparison to untreated control
Al-Samadi et al. (92)	A dynamic culture method as drug screening platform	-PDL1 antibody -IDO1 inhibitor	Single	**Primary:** Primary H&N squamous cell carcinomas, T cells, B cells, NK cells, monocytes, and dendritic cells **Cell lines:** -HSC-3	**2D:** Unexposed control Dynamic flow **3D:** Chambers coated with ECM substitute Dynamic flow Unspecified pump	NS	-Migration assay -Immunofluorescence staining - CellTiter 96^®^ Proliferation Assay -Cell Trace kit	-Preclinical organotypic model for personalized medicine and testing -IDO 1 inhibitor influences immune cell migration to cancer cells -Therapy response was reported to be patient dependent
Bower et al. (94)	A dynamic culture method as maintenance platform	NS	Single	**Primary:** Human biopsies of laryngeal, oropharyngeal, or oral cavity tumors staged at T2–T4 **Cell lines:** -NS	**2D:** Unexposed control Dynamic flow **3D:** Biopsy chamber Dynamic flow Syringe pump	NS	-H&E staining -Trypan blue -Flow cytometry -MTS proliferation assay	-Patient-derived samples were viable for 48 h after placement in the microfluidic chip -No significance difference concerning the average proliferation of samples pre- and postcultured in the chip
Lugo-Cintrón et al. (95)	A dynamic culture method as angiogenesis platform	NS	Two-culture	**Primary:** Human tubular lymphatic vessels and cancer-associated fibroblasts **Cell lines:** -HLEC2500 -HOrF2640	**2D:** Unexposed control Dynamic flow **3D:** Collagen hydrogel adhesion chamber Dynamic flow Syringe pump	NS	-H&E staining -Immunofluorescence staining -Immunohistochemistry staining -Proliferation assay -Migration and permeability assay -RT-qPCR -Multiphoton microscopy	-Preclinical organotypic model for personalized medicine and testing -Cancer-associated fibroblasts increased the gene expression of prolymphangiogenic factors in the lymphatic-like vessels
Sharafeldin et al. (96)	A dynamic culture method as biomarker detection platform	NS	Single	**Primary:** NS **Cell lines:** -HN12 -HN13 -HN30 -CAL-27	**2D:** Subtract biomarker control signal **3D:** Sonic-assisted chemical lysis chambers Dynamic flow peristaltic micropump	NS	-Biomarker quantification (desmoglein 3, VEGF-A,VEGF-C, β-Tub)	-Biomarker detection model for cancer metastasis diagnostic -Limit of detection was below 0.20 fg/ml of the analyzed analyte in 20-min evaluation
Jin et al. (103)	A dynamic culture method as a chemotherapy screening platform	-Paclitaxel -Cisplatin -5-FU	Two-culture	**Primary:** Patient-derived tumor cells from squamous cell carcinoma and salivary gland adenoid cystic carcinoma **Cell lines:** -ACC-M -UM-SCC-6 -HUVEC	**2D:** NS **3D:** Transwell-like channels -Bottom chambers coated with an ECM substitute Dynamic flow Double syringe pump	NS	-Hoechst 33342 and propidium iodide -Immunofluorescence staining	-Preclinical organotypic model for personalized medicine and testing -High concentration of drugs did not provide a therapeutic effect as HUVEC cells were killed. A lower concentration was recommended to provide a therapy to kill cancer cells (over 50% apoptosis) and low HUVEC cytotoxicity (over 50% viability) -Therapy response was reported to be patient dependent concerning different low drug concentrations

NS, not studied.

The first microfluidic device for H&N cancer drug screening was designed by Hattersley et al. *via* lithography in polydimethylsiloxane and a syringe pump (49). Primary H&N squamous cell carcinoma biopsies (~3-mm^3^ size) placed in the microfluidic device equipped with a syringe pump were exposed to cisplatin and 5-flourouracil continuous flow up to 7 days. Results showed decreases in cell viability and proliferation on drug-exposed groups compared to unexposed controls. In addition, the sandwich ELISA results of cytochrome c, a key compound in cell apoptosis, were found higher in the culture media in the treated groups compared to untreated controls. This study represented an important step of evaluating the personalized treatment of patient’s tumor biopsies under constant drug irrigation.

Riley et al. (91) further advanced the design of microfluidic platforms for personalized H&N drug screening. This platform was fabricated with two polyether–ether–ketone support plates, a silicone gasket as a tissue well, and a syringe pump. Such platform was applied to evaluate the effect of a combined JNK inhibitor and etoposide drug treatment on thyroid cancer biopsies (~5-mm diameter) from 23 individual patients. After 4 days of drug exposure, increased cell death was found in the thyroid cancer biopsy group compared to the unexposed group although no patient-specific drug responses were observed in this study.

Interconnected compartmentalization strategies within microfluidic devices for H&N cancer modeling were first implemented by Jin et al. (103). Their microfluidic platforms were made of two layers of polydimethylsiloxane interconnected by a porous polycarbonate membrane and flow applied *via* a double syringe pump. This membrane allowed the nutrient/drug exchange between the top chamber of endothelial cells Human umbilical vein endothelial cells (HUVEC) and the bottom chamber of cancer spheroids (103). To further optimize the device design, bubble trappers were proposed to facilitate continuous laminar flows and avoid chamber blockings in synchronous drug delivery, which is known prone to the bubble generation within microfluidic devices. This platform was also designed to emulate the tumor perivasculature by using concentration gradient chambers. These chambers comprised two drug inlets with six downstream channels for parallel drug gradient formation connected to the HUVEC culture chambers. Patient-specific or human salivary adenoid cystic carcinoma (ACC-M cell line) were used to fabricate cancer spheroids. Cell spheroids were subjected to parallel drug exposure mimicking the dual treatment of cisplatin/paclitaxel or cisplatin/5-fluorouracil *via* the two-drug inlet synchronous application. After a 24-h parallel exposure of combined drug treatments, cell viability ACC-M spheroids (ACC-2 group) showed higher sensitivity (i.e., more cell death) to cisplatin/5-fluorouracil treatment whereas patient-specific spheroids (SCC-1 group) were more sensitive to cisplatin/paclitaxel treatment.

## Future prospects

H&N cancer drug studies with microfluidic models emphasized the importance of using patient-derived biopsies from oral cavity, pharynx, larynx, lymph nodes, and thyroid for patient-specific prediction of drug response (49, 91, 94), echoing those as in the review of organoid models. Patient-derived tissue biopsies preserve key cellular heterogeneity and geometry of the tumor, which are important variables for drug screenings. However, the use of tissue/tumor biopsies for microfluidic platforms is hampered by the technical challenge of on-chip imaging and off-chip analysis (104). Milliscale tissues as tumor biopsies usually give raise to culture challenge concerning the complex tissue preservation during long-term culture times (105). Fortunately, advances in microfluidic platforms make the long-term culture of thick tissue samples possible with an effective nutrient and oxygen supply through a dynamic flow of culture medium (49, 91). In particular, pump-free microfluidic devices were shown to be able to maintain 2-mm human organotypic models for a 75-day continuous culture of human brain organoids (106).

**Figure 7:**
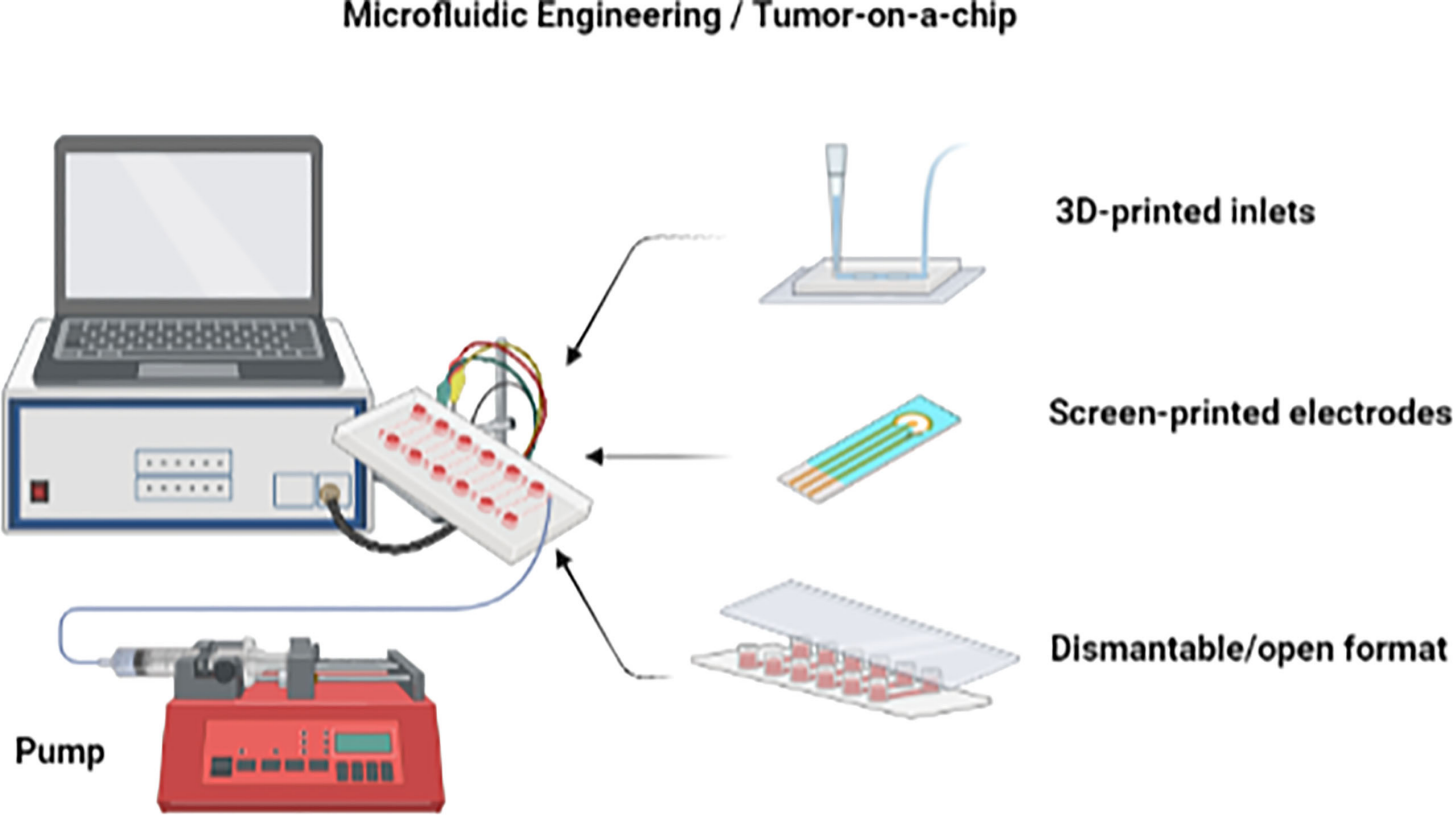
Advances in microfluidic technology. Microfluidic devices as tumor-on-a-chip may incorporate 3D-printed components and a dismantable/open format. Figure created with BioRender.com.

Other advances in microfluidic technology, such as dismantable/open and droplet-based formats, also facilitate the development of tumor-on-a-chip devices (104) (**Figure 7**). The dismantable/open-layer feature of microfluidic platforms allows for the direct retrieval of the analyzed samples by taking apart the top layer of the device (104). Cultured materials can then be easily accessible for off-chip analysis as the histological staining of biopsies and biopsy-like tissues. The fabrication of tumor-on-a-chip platforms can be complicated due to the necessity of having a microscale cell culture environment and chamber flow interconnection, which often requires high manual skill sets. The use of 3D printing for creating the on-chip microcomponents such as chambers, membranes, and grooves is therefore a very wise option to save labor and costs compared to conventional lithography and polydimethylsiloxane molding (107–111).

Lastly, combined chemotherapy drugs, namely, cisplatin and docetaxel, have already been tested as a tumor reduction strategy in HPV^+^ oropharynx cancer patients (112). The multicompartments of microfluidic devices can be harnessed for screening multiple therapeutics in parallel, mimicking various combinations of cancer drug treatments like dual chemotherapy drugs or even the combination of chemoradiotherapy (105).

## Future outlook

**Figure 8:**
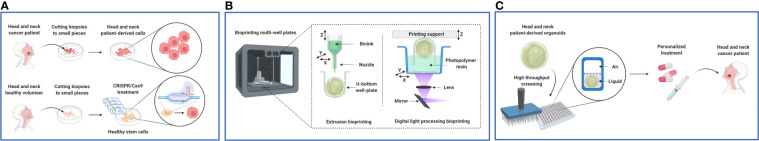
Future outlook of *in vitro* H&N cancer patient-derived organoid (PDO) models. **(A)** Sourcing of H&N PDO models using the tumor biopsies of cancer patients and CRISPR DNA–modified healthy cells. **(B)** Fabrication of H&N PDO models using bioprinting. **(C)** H&N PDO model life span used as air–liquid interface in HTS for personalized medicine purposes. Figure created with BioRender.com.

The development of multicellular tumor spheroid systems that are compatible for preclinical studies, as HTS drug screening (113), is one important milestone of advancing personalized cancer medicine (114). As a result, PDOs became increasingly used to preserve part of the structural features and genome, epitome, transcriptome, proteome, and metabolome information of an individual’s H&N tumorigenesis for anticancer drug studies (115–117). Certain challenges such as suboptimal reproducibility and high manufacturing costs are well-known barriers with advanced culturing systems. In particular, the development of microfluidic devices requires specialized microfabrication and operation skills. Below, we further present specific challenges with PDOs in their adaptation for HTS with respect to their sourcing, fabrication, and culturing life span (**Figure 8**).

## Overcoming the limited source of patient-derived organoids

Tumor tissue biopsies are needed from cancer patients to generate PDOs, but the source is often limited and unpredictable with clinical samples (**Figure 8A**). Fortunately, PDOs can be replicated and cryopreserved in specialized facilities, known as living biobanks, without losing cell-type specificity (87, 118). For example, intraductal papillary mucinous neoplasms were collected from patients with pancreatic cancer (119). The tumor tissues were first digested with a proteolytic enzyme for cell retrieval. The recovered cancer cells were then seeded in Matrigel^®^ and stored as PDOs in a living biobank (119). The gene analysis data of key markers KRAS, PTEN, PIK3CA, GNAS, RNF43, and BRAF showed a similar expression between PDO and the patient’s tumor tissue biopsy, which confirmed the preservation of patient samples’ genome in living biobanks.

The stock of PDOs from living biobanks can be further expanded with the method of patient-derived xenografts (PDXs) (120–122). A PDX is to first insert PDOs in animals and then amplify the PDOs within the host. The derived PDXs (i.e., cloned PDOs) are then cryopreserved and stored in living biobanks, preserving cell–cell interactions as those of parent tumor. Of note, the genome copy number alterations of PDX-expanded PDOs may change after extensive passaging due to possible host reactions to the implant (123–125). As such, if a high passage (>P10) is used in treatment, caution needs to be exercised as PDOs and PDX-expanded PDOs may display a differentiated response to drug therapeutics. Furthermore, PDX models are time consuming and expensive, the engraftment efficiencies may be different among the TME types, and finally, the immune response cannot be properly evaluated due to the immunodeficiency of host strains (126). As a result, additional cancer model strategies are thus required.

## Patient-derived organoids from cancer and healthy stem cells

In addition to tissue biopsies, organoids can be grown from cancer or healthy stem cells (115, 116, 120, 123, 127–133) although their use in cancer research is still in its infancy (128, 133–135). PDOs from cancer stem cells possess metastatic, chemotherapy, and radiotherapy resistance features, while healthy stem cells do not present those intrinsic characteristics (133, 136). At the same time, cancer stem cells are criticized of their limited clonal heterogeneity (133). A plethora of cancer-associated markers such as CD133, CD44, ABCG2, aldehyde dehydrogenase, octamer binding transcriptional factor 4, SOX2, and NANOG have been reported in cancer stem cells (134, 135, 137). However, marker expression does not necessarily translate into a cancer stem cell phenotype without transplantation assays (138). These assays are necessary to verify and characterize the tumor-initiating and -regenerating capabilities of such cells on implanted hosts.

Conversely, healthy adult stem cells like mesenchymal stem cells (139) and induced pluripotent stem cells (140) are another option of PDOs in cancer research. Human-induced pluripotent stem cells from healthy adults were proposed to generate PDOs for liver cancer studies (140). For instance, induced pluripotent stem cell reprogramming from human fibroblasts was successfully directed toward a hepatic endoderm-like phenotype *via* differentiation media containing activin A, bFGF, and BMP4 after 8 days of exposure (141). Then, the exposure of differentiation media with NOTCH activator agents to generate liver tumoroids or NOTCH inhibitors for liver organoids was performed after 2–3 weeks (140). The aforesaid methodology could be adjusted, following the generation protocol of vocal fold mucosae from human-induced pluripotent cells (142). At that point, the PDO fabrication protocol for H&N cancer may implement the upregulation of Snail, the downregulator of epithelial markers and the upregulator of mesenchymal markers (143), and exposure to FGFs to generate stratified squamous epithelia (139, 144).

In more detail, induced pluripotent stem cell–based cancer modeling can be used as follows (145): (I) genetic alterations can be engineered into normal human-induced pluripotent stem cells using transcription activator-like effector nucleases (TALENs) or CRISPR/Cas9 (146). These stem- derived cells with engineered cancer-associated mutations can be used to acquire the initial cancer molecular events to then emulate cancer progression (145). (II) Induced pluripotent stem cells can be used to reprogram patient-specific somatic cells with cancer predisposition syndromes such as Li–Fraumeni syndrome (147). (III) Induced pluripotent stem cells can be engineered as cancer-specific cells by targeting tumor suppressors such as SMAD4, Rb/P16, BRCA1, CDKN1A, and CDKN2A (145). The previously mentioned stem cell strategies may help advance PDO research on H&N cancer.

Lastly, human embryonic stem cells were implemented as organoids for metastatic brain cancer modeling using induced pluripotency stem cell strategy (148). However, the use of embryonic stem cells possess ethical concern, low immune compatibility and potential rejection after clinical transplantation (149). Nevertheless, continuous *in vitro* validation such as phenotype analysis is warranted to ensure the safe use of healthy stem cells as PDO models for cancer research.

## Patient-derived organoids from CRISPR/Cas9 DNA-modified healthy cells

CRISPR/Cas9 transgenesis technology has been proposed to genetically modify healthy biopsies into PDOs (115, 116, 120, 123). The technology of CRISPR/Cas9, simply put, involves activating/silencing a specific gene of target (**Figure 8A**). CRISPR/Cas9-mediated genome editing comprises the implementation of two components: (I) single-effector Cas9 protein to allow double-stranded breaks in the target DNA and (II) a single-guide RNA to guide the Cas9 complex to the targeted genomic zone (150, 151). The CRISPR/Cas9 technology has already been used to fabricate human oncogenic organoids from healthy liver by editing PTEN/TP53 and from healthy colon by targeting APC/SMAD4/TP53/K ras/PIK3CA (152). Interestingly, human pluripotent stem cells can gain CRISPR/Cas9-mutated p53 with a critical functional evaluation of p53 to avoid double-strand break toxicities dependent on p53/TP53 (153). Furthermore, wild-type human gastric organoid cell lines with ARID1A, an early-stage gastric cancer marker, as a single mutant target has been modified through CRISPR/Cas9 technology (154). In H&N cancer, gene editing may target the EGFR/PI3K/Akt/mTOR pathway for oncogenic organotypic fabrication.

One known limitation with CRISPR/Cas9 technology is related to the low specificities to the target genes (150, 152). For instance, the off-target effect is often observed at a rate ≥50% in RNA-guided endonuclease-induced mutations in unintended target zones (150, 155). *In silico* libraries as the sgDesigner tool can be used to optimize the design of novel plasmids by including both the single- guide RNA and the target site that was not used before (150). In addition, implementing Cas9 variants such as Cas9 nickase has also been used to induce single-stranded breaks combined with a single-guide RNA in order to produce double-stranded DNA breaks at the desired location (150).

## Overcoming the fabrication complexity of patient-derived organoids

Organotypic models provide a superior potential in patient-specific cellular heterogeneity, molecular phenotypes, tissue–stratum architecture, and geometry (156). Bioprinting may help fabricate PDO fabrication in a more precise and automated manner compared to conventional PDO production. Specifically, the layer-by-layer strategy of bioprinting can help to generate spatial-specific cell distribution and ECM architecture in PDO fabrication (**Figure 8B**). This strategy is empowered by inkjet/extrusion, laser-assisted, and stereolithography bioprinting methods (120, 157–159). For example, a 3D digital light processing bioprinting/HTS study was conducted to bioprint hepatocellular carcinomas and HUVECs in 96-well plates (160). The bioprinted gelatin methacryloyl (GelMa)–based construct had the dimensions of 2.4 mm × 2.4 mm × 250 µm, highlighting the spatial precision of Digital light processing bioprinting (DLP) technology required for HTS.

Digital light processing bioprinting technology has enhanced the resolution (~10 times) of bioprinted PDOs, which has been one notable barrier with nozzle extrusion (159–162). In addition, digital light processing bioprinting offers a shear stress–free advantage over extrusion bioprinting by reducing potential cell damage during organotypic assembly (66, 158, 162). This shear stress–free printing method achieves a cell viability of ≥90% within the 3D-printed construct, whereas that of extrusion bioprinting is 40%–80% (66). Concerning multiple gradients in the printed assembly, digital light processing bioprinting presents a dynamic gradient tunability needed for proper recapitulation of complex anatomical structures compared to that of extrusion bioprinting (163). In addition, a low amount of bioink waste is found while changing the gradients using digital light processing bioprinting combined with microfluidic technology (163). Digital light bioprinting also allows to swiftly produce photopolymerized 3D constructs *via* a projected light (66, 160–162) (**Figure 8B**).

Typically speaking, the resolution of digital light processing bioprinting meets the need of organoid bioprinting (158, 159, 161). For instance, the resolution for inkjet/extrusion and laser-assisted bioprinting is ~50–500 µm and ~100 µm, respectively, while digital light processing bioprinting can achieve as high as 50-µm resolution (161, 162, 164). In general, digital light processing bioprinting take up to 40 min to entirely bioprint a 96-well plate (160) at the speed of 0.5–15 mm/s (164). Extrusion bioprinting has been reported to have longer fabrication times, 10–50 μm/s (165), because of the interaction between the bioink viscoelasticity and the extrusion nozzle size (166, 167). Given that the resolution necessary for the cell-laden tumor organotypic models is below 100 µm (161), the high-resolution capability of digital light processing bioprinting will allow precise fabrication of H&N PDO models without comprising the time cost. The increased resolution of 3D-printed organ-on-a-chip can also benefit the development of HTS platforms down the road (168).

The challenges of digital light processing bioprinting are the scarce number of photoinitiators such as Eosin Y, Irgacure 819, and lithium phenyl-2,4,6-trimethylbenzoylphosphinate (160, 164) and photo-crosslinking resins like GelMA, methacryloyl hyaluronic acid, and poly(ethylene glycol) diacrylate (159, 162, 164). A deficient concentration of photoinitiators within the construct provokes poor mechanical properties affecting the desired resolution and cell viability (159, 162). Because of that deficiency, proper standardization to balance the photoinitiator and resin concentrations will need to be carried out to achieve the reported cell viability ≥90% (66) and high resolution ≤50 μm (159, 162, 164). Another hurdle of digital light processing bioprinting is the limited incorporation of multiple materials within the 3D cell–based construct (159, 162, 164). However, digital light processing bioprinting can incorporate a multimaterial structure combined with microfluidics to print multiple bioinks (163). Taken into consideration the bioink component accessibility, nozzle extrusion bioprinting remains the most popular bioprinting method for bioprinting (159, 167, 169).

## Overcoming the long-term culturing of patient-derived organoids

Microfluidic chips allow the long-term culture of sizable biological micro-/milliscale samples such as PDOs with effective nutrient/waste exchange *via* the dynamic liquid flow within the chip (109). Recent airway-on-a-chip microfluidic platforms, especially those with air–liquid interface feature (170–173), are particularly suitable and adapted for H&N cancer modeling given that the H&N squamous cell carcinomas are constantly exposed to air. However, most airway-on-a-chip devices need pumps to perfuse air and liquid through the air–liquid interface channels, respectively (171–174). This pump requirement presents a critical challenge for the adaption of HTS arrays. To date, non-microfluidic air–liquid interface platforms may incorporate up to 96 individual Transwell plates (175), whereas microfluidic-based air–liquid interface systems are able to integrate up to 64 individual chambers at most (176). None of these are truly considered as high throughput, in which HTS is commonly known as testing hundreds of samples on one array.

That said, one most recent microfluidic platform, developed by Bircsak et al. (177), allowed to house tumor organoids cultures up to 200 individual chambers. This device comprised the use of a multiplexer fluid control, a perfusion rocker platform, and culture chambers overlayed by the three-lane fluid channels. One of the analyses of this liver-on-a-chip platform was to study the drug metabolism of five drugs: phenacetin, coumarin, diclofenac, terfenadine, and phenolphthalein. Adopting such a microfluidic platform with air–liquid interface and pump-free features will present a great leap of advancing in *in vitro* H&N cancer modeling for high- throughput drug screening (**Figure 8C**). Accomplishing the combination of human multiorgan-on-chips (178) and high-throughput testing could benefit personalized anti-cancer therapy screening and discovery to boot (179).

## Conclusion

Geometry, multicellularity, and constant irrigation are key features for developing H&N-specific *in vitro* models for drug screening and discovery. Organotypic multicellular spheroid and organoid cultures are highly applicable to approximate cancer-specific TME by mirroring desired geometry and cell–cell/–ECM interactions as presented *in vivo* tumor tissues. Organotypic models can be further combined with microfluidic devices to evaluate the crosstalk between cells and barriers to the mass transport of oxygen, nutrients, and drug therapeutics. Ultimate *in vitro* H&N models can be achieved by incorporating PDOs, air–liquid interface, and high-throughput readouts for *de novo* oncology drug discovery and evaluation. The adoption of such a tumor-on-a-chip platform is expected to minimize the need of animal models and reduce the chance of failures in clinical trials for translational research.

## Authors contributions

CM-G: Conceptualization, visualization, writing-original draft, reviewing and editing. NL-J: Conceptualization, visualization, writing-original draft, reviewing and editing, supervision, funding acquisition. MT: Conceptualization, visualization, writing-original draft, reviewing and editing, supervision. HO: visualization, writing-reviewing and editing. NS: writing-reviewing and editing. JL: writing-reviewing and editing. All authors contributed to the article and approved the submitted version.

## Funding

This study was supported by the National Sciences and Engineering Research Council of Canada (RGPIN-2018–03843 and ALLRP 548623-19; PI: N.L.-J.), Canada Research Chair research stipend (J.Y.L., M.T., N.L.-J.) and the National Institutes of Health (R01 DC-018577-01A1). The presented content is solely the responsibility of the authors and does not necessarily represent the official views of the above funding agencies.

## Acknowledgments

We thank Dr.S. Yo Kishimoto and HO’s team for their work in obtaining permission for the clinical figure.

## Conflict of interest

The authors declare that the research was conducted in the absence of any commercial or financial relationships that could be construed as a potential conflict of interest.

## Publisher’s note

All claims expressed in this article are solely those of the authors and do not necessarily represent those of their affiliated organizations, or those of the publisher, the editors and the reviewers. Any product that may be evaluated in this article, or claim that may be made by its manufacturer, is not guaranteed or endorsed by the publisher.

## Abbreviations

2D, two dimensional; 3D, three dimensional; 5-FU, 5-fluorouracil; CCL-2, C–C motif chemokine ligand 2; ECM, extracellular matrix; EGFR, epithelial growth factor receptor; EMT, epithelial–mesenchymal transition; FDA, Food and Drug Administration; H&N, head and neck; HGF, hepatocyte growth factor; HPV, human papillomavirus; HTS, high-throughput drug screening; IL, interleukin; LTS, low-throughput drug screening; MDSC, myeloid-derived suppressor cell; MEK, mitogen-activated protein kinase; MMP, metalloproteinase; mTOR, mammalian target of rapamycin; PD-1, programmed cell death 1; PDO, patient-derived organoid; PDX, patient-derived xenograft; R&D, research and development; TGF, transforming growth factor; TME, tumor microenvironment; UV, ultraviolet; VEGF, vascular epithelial growth factor.
